# Promoting Physical Activity and Reducing Sedentary Behavior in Disadvantaged Neighborhoods: A Qualitative Study of What Women Want

**DOI:** 10.1371/journal.pone.0049583

**Published:** 2012-11-14

**Authors:** Megan Teychenne, Kylie Ball, Jo Salmon

**Affiliations:** Centre for Physical Activity and Nutrition Research, School of Exercise and Nutrition Sciences, Deakin University, Burwood, Victoria, Australia; Pennington Biomedical Research Center, United States of America

## Abstract

Since women living in socioeconomically disadvantaged neighborhoods are more likely to be physically inactive and engage in higher levels of sedentary behavior than women living in more advantaged neighborhoods, it is important to develop and test the feasibility of strategies aimed to promote physical activity and reduce sedentary behavior amongst this high-risk target group. Thirty-seven women (aged 19–85) living in a disadvantaged neighborhood, and five key stakeholders, received a suite of potential intervention materials and completed a qualitative questionnaire assessing the perceived feasibility of strategies aimed to increase physical activity and reduce sedentary behavior. Thematic analyses were performed. Women perceived the use of a locally-relevant information booklet as a feasible strategy to increase physical activity and reduce sedentary behavior. Including weight-loss information was suggested to motivate women to be active. Half the women felt the best delivery method was mailed leaflets. Other suggestions included reference books and websites. Many women mentioned that an online activity calendar was motivational but too time-consuming to commit to. Most women preferred the information booklet as a strategy to increase physical activity/reduce sedentary behavior, yet several suggested that using the booklet together with the online calendar may be more effective. These findings make an important contribution to research informing the development of intervention strategies to increase physical activity and reduce sedentary behavior amongst women living in disadvantaged neighborhoods.

## Introduction

Participating in physical activity [1] and engaging in lower levels of sedentary behavior [2] (i.e. sitting behaviors that expend very low levels of energy (1–1.5 METS) [3]) have been found to be independently associated with a lower risk of chronic diseases such as obesity, type 2 diabetes, and cardiovascular disease. Women living in socioeconomically disadvantaged neighborhoods are at an increased risk of physical inactivity [4], [5] and also of engaging in greater amounts of sedentary behavior [6], [7], independent of individual level socioeconomic indicators. For example, those living in the most disadvantaged neighborhoods have been found to spend on average 28 hours per week engaged in screen time (i.e. television viewing and computer use - the most common leisure-time sedentary behaviours), which is an additional 7 hours per week compared to those living in neighborhoods of high socioeconomic position [6]. These inequities may be partly explained by disadvantaged neighborhoods having poorer urban design [5], poorer accessibility of facilities and higher perceived levels of crime [8] compared to more advantaged neighborhoods. Since inactivity-related illnesses have also been linked to living in socioeconomically disadvantaged neighborhoods [9], [10], [11] it is important to identify effective intervention strategies to increase physical activity and reduce sedentary behavior amongst this high risk group.

Little research has aimed to investigate the most effective strategies to increase physical activity interventions amongst women living in socioeconomically disadvantaged neighborhoods [12], and of the existing intervention evidence in this and other disadvantaged target groups, most studies have focussed on women in the United States from racial/ethnic minority backgrounds [13]. Further, although print-based materials have generally been useful as support strategies for increasing physical activity in adults [14], the feasibility of print-based materials to increase physical activity specifically amongst women living in socioeconomically disadvantaged neighborhoods is not known.

There has been increasing interest in recent years in web-based physical activity interventions [15] with several being found to be successful in increasing women’s physical activity levels [16], [17]. Although programs such as the Women’s Active Living Kits (WALK) Pilot Program [18] have used web-based components to promote physical activity amongst ‘priority groups’ of women (including women with young children, older women, and culturally and linguistically diverse groups), to date, no web-based physical activity interventions have been designed specifically for, and assessed among women living in socioeconomically disadvantaged neighbourhoods. Very few intervention strategies have aimed to reduce sedentary behavior (as opposed to only increasing physical activity) in adults, let alone women living in socio-economically disadvantaged neighborhoods [19], [20]. Of the small number of intervention studies that have targeted reductions in sedentary behavior amongst adults, a large proportion included strategies that were predominantly focused on changing physical activity behavior [21], [22]. To our knowledge, no previous studies have tested the perceived feasibility and effectiveness of intervention strategies aimed at increasing physical activity and reducing sedentary behavior amongst women living in socio-economically disadvantaged areas.

This study aimed to investigate the perceived feasibility of two proposed intervention approaches (one print-based and one web-based) designed to promote physical activity and reduce sedentary behavior amongst women living in socio-economically disadvantaged areas. Further, the study aimed to investigate the feasibility of the two proposed intervention strategies amongst a small sample of key stakeholders representing public policy, health promotion, and community health and well-being. The development of the intervention strategies in this study were informed by a previous study of women living in disadvantaged neighborhoods with depressive symptoms, whereby women identified several potential strategies that they felt would help increase their levels of physical activity and reduce sedentary behavior [23]. Potential strategies included: the provision of information on available facilities to be active in the neighborhood; wider variety of classes/facilities and social exercise groups; awareness-raising of the benefits of physical activity; learning time management skills; provision of childcare facilities (e.g. more one-hour care facilities); and new mother’s exercise groups. Similar strategies (e.g. information/education, variety of activities/classes and social activities) have also been previously suggested by ‘priority groups’ of women who had participated in the WALK Pilot Program [24]. Since qualitative methods are useful for investigating areas in which little is known, the qualitative design of the current study was selected in order to provide rich insights for the development of feasible strategies to promote physical activity and reduce sedentary behavior amongst women living in disadvantaged neighborhoods.

## Methods

### Ethics Statement

This study was approved by the Deakin University Human Research Ethics Committee. Written consent was obtained from all participants.

### Participants

During May 2010, participants were randomly recruited from one urban Victorian neighborhood of low socio-economic position, based on the Australian Bureau of Statistics - Socioeconomic Index for Areas [25]. Address and neighborhood data were obtained using the spatial data files VicMap Address and VicMap Admin. Residential addresses within the selected neighborhood were identified and extracted using Geographic Information System (GIS) software (ESRI: ArcGIS 9.2. Redlands, CA 2007). The database enclosing all residential addresses for the selected neighborhood was used to randomly select 850 households.

Qualitative questionnaires were mailed to those 850 households and women living in a selected household and aged 18 or over were invited to participate. Where there was more than one eligible woman living in a household, the woman with the next birthday was asked to participate. Furthermore, two local community/neighborhood houses agreed to display recruitment posters with contact information for the study on their noticeboards. From the 850 qualitative questionnaires sent out, a total of 67 were classified as return to sender (due to insufficient address); 37 women returned a completed qualitative questionnaire representing a response rate of 5%. The recruitment posters in the community/neighborhood house/centre did not result in any contacts from women. Since socioeconomically disadvantaged groups are typically harder to reach, rather than require a multi-step approach to recruitment (e.g. advertise, require a response by participants in order to then be sent qualitative surveys, etc), we tried to make it a simpler approach by providing all the required study materials in a single step so they could see immediately what was involved and that participating only involved one step/response.

A second group of participants, consisting of representatives from stakeholder organisations within the same neighborhood whose roles included public policy, health promotion, or community health and well-being were also selected to participate in the current study (n = 8). Of the eight key stakeholders selected and contacted, seven agreed to participate with five returning a completed qualitative questionnaire (recruitment details are described below).

### Recruitment

Households selected for the study were sent a survey pack in the mail, including a letter informing recipients that their household had been selected to take part in the study. The pack also included two pilot intervention strategies (one print-based information booklet and one web-based activity calendar with instructions), a qualitative questionnaire and a reply-paid envelope. Following the Dilman protocol [26], a reminder letter was mailed out to selected households two weeks later. Key local stakeholders within the study neighborhood were identified from organisations (local council, health promotion organisations, and neighborhood/community houses) that had direct involvement in promoting health and wellbeing in the community. A letter of invitation was sent to seven key stakeholders and followed up with a telephone call two weeks later. Stakeholders who agreed to participate were then sent a survey pack in the mail.

### Intervention Materials

Both interventions were developed incorporating elements from the social ecological model [27] (i.e. intra-personal, social and environmental factors such as increasing knowledge, providing opportunities for social support and information on available physical activity facilities in the area) as well as successful behavior change strategies (e.g. goal-setting, self-monitoring) identified in reviews including adults [28], [29] and previous studies amongst women [17], [30]. A previous qualitative study by Teychenne *et al*. [23] further informed the conception and development of strategies to be tested.

#### Information booklet

The information booklet was a 15-page locally relevant booklet designed and tailored specifically for the selected study neighborhood. It provided information about why it is important to be physically active and reduce sitting time, practical ideas to increase levels of physical activity and reduce sitting time, availability of facilities to exercise in the neighborhood (including women-only facilities, recreational clubs, places to walk your dog, exercise options for new mothers, as well as safer areas to exercise), and availability of childcare facilities in the specific study neighborhood. The booklet was designed to require minimal reading, used simple language and was visually appealing (e.g. included numerous pictures of the local area).

#### Online activity calendar

The online activity calendar was a print off of a ‘mock’ website designed to allow users to log on and schedule their physical activity goals for the week, month and year. The site was designed such that the user could mark off daily when they had been physically active and/or reduced time spent sitting. At the end of the week, the computer program was designed to determine whether the user achieved their physical activity goals and it provided tailored feedback for the user based on actual physical activity levels. There was also an online forum in which it was proposed users could chat with other women and share any tips and advice they have for becoming physically active and reducing sitting time.

### Measures

The qualitative questionnaire included questions which required participants to provide written feedback on their views of the potential effectiveness and feasibility of two proposed intervention strategies provided in the survey pack. Furthermore, the qualitative questionnaire for women included self-report measures of socio-demographic characteristics (age, marital status, children living at home, education, employment status, and country of birth), physical activity, and sedentary behavior.

#### Physical activity and sedentary behavior

Women’s leisure-time physical activity was measured for descriptive purposes only, using items from the International Physical Activity Questionnaire (IPAQ-L), a validated measure involving a seven-day recall of physical activity behaviors [31]. The reliability of the IPAQ-L has been tested and has comparable validity to most other established self-reported physical activity methods [31], [32]. Four measures of sedentary behaviors involving a seven-day recall were also included in the qualitative questionnaire for descriptive purposes only: overall time spent sitting at work, overall time spent sitting during leisure-time [31], time spent sitting watching television [33] and time spent sitting at a computer [33].

#### Perceived feasibility of intervention strategies

The perceived feasibility of the two potential intervention strategies was assessed through a series of short-answer, open-ended questions. Questions included: “What do you like about the information booklet?”, “What don’t you like about the information booklet?”, “What other information do you think would be useful in motivating you to be more active?”, “What other information do you think would be useful in motivating you to sit less?”, “In what way would the information be best delivered?” and “Which strategy do you think would be more likely to result in an increase in physical activity and a reduction in daily sitting time, and why?” Key stakeholders were provided with the same questions in regards to their perspectives about what they felt would be most feasible for women in their community.

### Data Analysis

Data were analyzed using a thematic data analysis method adapted from Braun & Clarke [34]. After repeated reading of all short-answer responses, hand-coding of the qualitative questionnaires was conducted by creating and assigning descriptive labels to parts of the text. Following this, the qualitative data analysis program NVivo was used to organise data and facilitate the creation of categories and sub-categories and identify relevant quotes. For this study, the NVivo coding structure created was relatively simple. For each question, categories were created for each participant group (i.e. women versus key stakeholders) through the grouping of codes (sub-categories) that shared similar content about each strategy. Key themes were then identified. Furthermore, for those questions on ‘which approach you would prefer?’, simple counts were taken and tallied. In order to test the comparability of the interpretation of the data and ensure results were not subject to researcher bias, researcher triangulation was employed [35] whereby all qualitative questionnaires were coded by one author then a random subset of four questionnaires was independently cross-coded by a second author and third author. No discrepancies in coding or interpretation were observed. Written quotes were selected to illustrate key themes and are presented with the participant’s age and assigned pseudonym.

## Results

The final sample consisted of 37 women and five key stakeholders. The mean age of participants was 50 years. Two-thirds of the sample were in a married or defacto (i.e. living with their partner) relationship (65%) and 40% of women had children living at home. Just over half of the women were employed part-time or full-time with just under one-quarter of women retired (22%). About 10% reported a low level of education (had not completed secondary education), 38% a medium level (completed year 12 and/or vocational training), and half had a university degree (51%). All five key stakeholders were women who worked in managerial roles representing local government, a neighborhood/community house, a local gym, a specialised personal training business, and a community health centre.

Of the physical activity undertaken in leisure-time, women spent on average 2.75 hours walking in the last 7 days and 1.75 hours in other moderate and vigorous-intensity physical activity in the previous week. Participants reported approximately 31 hours in the previous week sitting during leisure-time (∼4.5 hr/day).

### Information Booklet: What Women Liked

On balance, most women and key stakeholders were impressed with the booklet and suggested that it was something that they would be using for future reference.

*“Can I please be subscribed to it!”* (Erin, 21)

#### Informative

Nearly every woman mentioned that they liked the level of detailed information included in the booklet. However, the type of information seen as most important varied for women. Some mentioned liking the inclusion of prices, times and contact information for clubs and facilities, while others mentioned that they liked that it exposed them to a variety of activities that they were unaware of.

*“It is a comprehensive booklet giving advice on ways to keep fit as well as giving details of lots of activities to get involved in”* (Wilma, 63)

It was apparent that women appreciated that all the information was sourced for them, saving them time and effort when preparing to be physically active.

*“I think it is a great way to see everything that is out there for women of all ages and abilities without having to do the searching”* (Fiona, 56)

#### Local relevance

It was evident that women living in the selected neighborhood felt that they had previously received little attention in regards to being provided local health and physical activity information. Several women mentioned appreciating how locally relevant the information in the booklet was.

*“Having just moved to Neighborhood X I loved the book. I just didn’t realise there was that much to do”* (Katrina, 56)

#### Presentation

Over half the women mentioned that the booklet was comprehensive and well presented, particularly remarking about the colours and photos included. This was further highlighted by several key stakeholders.

*“The booklet was well laid out and very easy to read”* (Irene, 54)

#### New ideas to increase physical activity and reduce sedentary behavior

It was apparent that a number of women appreciated the way that the information booklet was able to provide ideas on how to fit physical activity into busy women’s lives. Similarly, a couple of woman mentioned that the tips to reduce sedentary behavior were useful and that they would start implementing some of these strategies.

*“Nice suggestions for “life organisation” to fit exercise in to “time-poor” women”* (Ingrid, 55)

### Information Booklet – What Women did not Like

#### Lack of personal tailoring

A number of women suggested that the information booklet lacked information relevant to their personal needs and lifestyles. For example, several women mentioned that the times of exercise classes that were referred to in the information booklet did not fit in with their weekly schedule due to work or family commitments.

*“Many of the activities are during my working hours and therefore I’m not likely to be able to make it”* (Nicole, 30)

Similarly, a few older women suggested that the booklet needed to include more information on exercise options that were relevant to their age-group.

*“Perhaps “age-specific” activities, clubs, groups would be more interest to me, as I am 57 years old and have some illnesses to contend with at present”* (Pearl, 57)

#### Unrealistic tips

Several women thought that the booklet provided unrealistic tips on increasing physical activity and reducing sedentary behavior. This view was further highlighted by a key stakeholder.

*“Having seen the number of overweight women in Neighborhood X, it may be a tall order for many to consider getting up 30 minutes earlier, a more realistic suggestion may be 15 minutes”* (Edith, 67)

### Other Useful Information to Increase Motivation for Physical Activity

A number of suggestions were proposed by women that may help to motivate them to be more physically active. These included being provided with information tailored to the individual (i.e. age-group relevant exercise classes/groups) as well as other suggestions not previously described.

#### Weight loss information

It was apparent that women were particularly interested in physical activity for the purpose of losing weight. Therefore, several young women suggested incorporating other weight-related information (e.g. nutrition) into the booklet to help women with weight management.

*“Being told [that] if I did something extra, I’d lose so many kg’s”* (Bridie, 36)

*“Can add healthy eating/seasonal recipe ideas”* (Nicole, 30)

Similarly, this kind of approach (providing weight loss information) was suggested by one key stakeholder in order to reduce sedentary behavior.

*“People like info i.e. If you get up and change the channel rather than use the remote you can burn 20 calories each time. Weight loss always motivates women”* (Key Stakeholder)

#### Seasonal suggestions

One woman mentioned that she would like to see more tips/suggestions in the booklet about exercises that you can do in winter in order to overcome such barriers.

*“More winter exercise ideas – it’s dark when I get home after work and very cold currently”* (Nicole, 30)

#### Vouchers

One key stakeholder suggested that to overcome the barrier of cost, and perhaps increase motivation to begin exercising, it may be useful to include discount vouchers in the booklet to the facilities or clubs within in the community e.g. gym discount.

*“I think you have provided all possible info but maybe including some vouchers from the companies you have put into the booklet”* (Key Stakeholder)

### Other Useful Information that may Help to Reduce Sedentary Behavior

Few women were able to suggest ideas on other information that may help them reduce their sedentary behavior (sitting time), with most reiterating their ideas on increasing physical activity. Suggestions offered were for further information that was already included in the booklet (i.e. health risks of sitting, and tips to reduce sitting).

*“More ideas on how to do things standing up”* (Bannie, 55)

*“Maybe more [information on] medical problems/issues arising from sitting too much”* (Rachel, 45)

### Information Booklet – Best Delivery Method

More than half the women felt that the information booklet would be best delivered through monthly leaflets in the mail.

*“Mail newsletter! Not everyone (including me) has easy access to e-mail or internet, or doesn’t have an I-phone. All at once would be too much info but monthly would be good”* (Erin, 21)

However, a few women suggested that the most appropriate way of delivering the material in the mail would be all at once to be used as a reference book, with one woman suggesting leaflets would only be thrown away.

*“Probably as a reference booklet to keep near the telephone and a point of reference for others in need”* (Edith, 67)

Alternatively, just over a quarter of women suggested that the information would be best delivered through a website or e-mail program, with a handful of women suggesting an i-phone application would be of value.

*“Via e-mail or i-phone application – better environmentally”* (Jacqui, 36)

A combination of mail, online technology (website or e-mail) as well as phone technology was suggested by a few women in order to distribute the information by tailoring the approach to the needs and lifestyles of all women. This was reinforced by the views of key stakeholders.

***“*** *All of the above* ***–*** *Take into account lower incomes (need leaflets), older adults (no computer), modern mums (X Gen, I-phone)”* (Key Stakeholder)

### Online Calendar: What Women Liked

#### Presentation/Easy to use

Most women mentioned that the online activity calendar was well presented and particularly liked the simplicity of the layout. Moreover, women suggested that the program was easy to understand and user-friendly, which appeared to be an important factor for key stakeholders also.

*“Well thought through. Easy to read. Easy to operate. Good layout”* (Hilary, 44)

#### Self monitoring/planning

A large proportion of women and key stakeholders mentioned that the online activity calendar was a good way to plan and track physical activity goals, with some women suggesting it would work as a ‘reminder’ to exercise.

*“I’m a schedule-loving soul so right up my alley”* (Verity, 64)

#### Motivational

A number of women believed the online activity calendar could be used as a motivational tool and that it was encouraging and somewhat rewarding. This view was replicated by key stakeholders.

*“An incentive to reach your goals. A reward when you do”* (Adelle, 58)

#### Tailored to the individual

A couple of women also suggested that they liked how the online activity calendar was personalised and tailored to individuals, which was reinforced by a key stakeholder.

*“Very personalised. Lots of positive reinforcement”* (Key Stakeholder)

### Online Calendar: What Women did not Like

Although only seven women said that they would not use the online activity calendar, most women suggested that there were aspects of the calendar that they did not like.

#### Time-consuming

Many women believed that the activity calendar would be energy- and time- consuming, with most women suggesting that they already lacked time in their lives and therefore logging on to this program may be seen as a chore.

*“That it’s another chore to add to already too many I have”* (Rachel, 45)

#### Goal non-achievement

A few women mentioned their concern about how they would feel if they did not achieve their physical activity goals for the week, suggesting that the online activity calendar may have negative effects on their motivation and achievement.

*“If you don’t reach goals at end of week could be depressing – Think people would tire of doing this every week”* (Fiona, 56)

#### Condescending tone

Several women felt that the online activity calendar was condescending; in particular the feedback provided when one achieved or did not achieve their weekly goals. A couple of women also suggested that self-motivated women may actually resent the program.

*“A bit condescending I think. Common sense – shouldn’t need to log-on etc to get motivated”* (Ingrid, 55)

### Other Suggestions to Improve the Online Activity Calendar

#### Weight loss information

Several women suggested that the online activity should provide weight loss information and tips, including information on nutrition as well as a calorie counter.

*“Tips on nutrition and diet”* (Wilma, 63)

Additionally, one key stakeholder suggested that the online activity calendar should include a weight tracker in order to monitor women’s weight loss throughout the year.

*“Could have a weight tracker which graphs weight”* (Key Stakeholder)

#### Information on physical activity ideas and facilities

A number of women felt that the online activity calendar should include a section with physical activity ideas and tips for example on overcoming barriers to physical activity, as well as information on local facilities and events, similar to the information provided in the information booklet.

*“A section with loads of ideas of things to do in various situations e.g. bad weather, injury, recovery from illness, no money”* (Bannie, 55)

#### Link to existing diary system

A couple of women suggested linking the online activity calendar to an i-phone application or to a system which people already use (e.g. Microsoft outlook), in order to minimise the number of programs women have to log onto each day.

*“Link it to an existing diary system perhaps an application on i-phone or attachment for outlook or similar”* (Nicole, 30)

### Preferred Strategy

The information booklet was by far the preferred strategy for the majority of women. Reasons for this included: women’s enjoyment of reading; the fact that is was less time-consuming than the online calendar; and the provision of a range of useful information and tips to be active.

*“The booklet is better for me as it is not as demanding nor exhortative as on-line diary/calendar would be”* (Hannah, 45)

However a large proportion of women and key stakeholders also suggested that using the information booklet in conjunction with the online activity calendar would be most preferred since each strategy is quite different.

*“BOTH – I think they go hand-in-hand with each other. The booklet lets you find out activities you can do and the diary/calendar motivates you to uphold your choices and plan”* (Orla, 62)

## Discussion

The aim of the current study was to investigate the perceived feasibility of two proposed intervention approaches (one print-based and one web-based) designed to promote physical activity and reduce sedentary behavior amongst women living in socio-economically disadvantaged neighborhoods. Furthermore, the study aimed to investigate the feasibility of the two proposed intervention strategies amongst a small sample of key stakeholders.

The information booklet was perceived by women and key stakeholders as a potentially feasible strategy to increase physical activity and reduce sedentary behavior. Since living in socio-economically disadvantaged neighborhoods is generally associated with lower education (including literacy) levels [36], the information booklet used in the current study was designed to ensure good readability, with the use of simple language and pictures. Most participants suggested that the information booklet was easy to read/understand and well presented. Self-instructional reading (information) materials have been suggested to be an effective and cost-effective strategy for increasing walking amongst disadvantaged women [37], provided that information is presented in a comprehensible style.

A common theme that emerged from the current study was the women’s desire for weight loss tools and information (e.g. diet tips, calorie counters) to be included within both the information booklet as well as the online activity calendar. Since women living in socio-economically disadvantaged neighborhoods are at a greater risk of overweight and obesity than women more advantaged neighborhoods [9], this may be an important component that could provide additional motivation in interventions amongst these women. A number of information-based weight-loss interventions in the general population have been effective in increasing physical activity [38] as well as facilitating weight loss [39] which provides further impetus for testing such strategies within a sample of women living in disadvantaged neighborhoods.

A small number of women suggested that the booklet lacked information relevant to their personal needs, age and/or lifestyle. Individual tailoring of information has been found to be a successful strategy for increasing physical activity as it increases the personal relevance of physical activity interventions for various sub-groups [40]. The findings of the current study indicate that tailoring booklets to various age-groups may be more appropriate. Nonetheless, exhaustive searches of local facilities were conducted, and to our knowledge, all local physical activity recreational clubs, classes and facilities were included in the booklet, suggesting that there may be a real lack of access and opportunities for older and working women to be physically active in the targeted neighborhood. Consistent with previous research [41] suggesting that community-wide physical activity promotion strategies are more likely to be successful when tailored to the attributes of target groups, findings of this study suggested that tailoring delivery methods of the booklet to suit different women’s needs, lifestyles and ages may also be important.

Several women and key stakeholders suggested that the tips provided to reduce sedentary behavior, such as standing at your computer, were impractical and unrealistic. Further, very few women were able to suggest ideas on other information that may help them reduce their sedentary behavior. The concept of reducing sedentary behavior for health benefits is a relatively new area of research and public health focus, and therefore a large proportion of the population may be unfamiliar with it. It is therefore important to promote more broadly the independent health risks linked to engaging in long periods of sedentary behavior, for instance through large scale mass media and social marketing campaigns.

The information booklet was perceived as the preferred and most feasible strategy for increasing physical activity and reducing sedentary behavior amongst the women. However, a number of women saw the value in both intervention strategies and felt that both the information booklet and the online activity calendar should be used together and would complement each other. Previous research comparing print and web-based physical activity interventions has found both strategies to be effective in increasing physical activity in adults [42]. However, one intervention study in the general population [43] reported participants receiving a print-based strategy increased their physical activity levels, yet participants receiving a web-based strategy decreased their sedentary behavior at the end of the study. Consistent with previous recommendations for children and adults [29], the findings of our study suggest that a multi-component approach may be the most successful method to increase physical activity and reduce sedentary behavior amongst women living in disadvantaged neighborhoods.

A major strength of the current study was the qualitative design, which provided detailed insights not possible from quantitative approaches. Furthermore, the inclusion of women from socio-economically disadvantaged neighborhoods in this study provided novel insights in an important group at risk of physical inactivity [44], [45]. Moreover, the current study tested the feasibility of ‘minimal’ intervention strategies that may be widely distributed to large numbers of people, as well as being relatively inexpensive and non-intensive [37]. Thus, these strategies may be more appropriate to implement within socioeconomically disadvantaged communities than more expensive or intensive strategies.

When interpreting the results of this study, a number of limitations should be considered. Firstly, socio-economic disadvantage was defined using an area-based measure. Therefore, a number of participants may not be considered to be socio-economically disadvantaged based on individual level indicators such as income or education. Nevertheless, area-based indicators of disadvantage have been linked to lower leisure-time physical activity, independent of individual socio-economic indicators [5]. Secondly, a number of the women in the current study were already quite active. The recruitment of an inactive sample may have yielded different results. Finally, the current study initially targeted a high-risk and typically hard to reach group (women living in socio-economically disadvantaged neighborhoods). Therefore, it was not surprising that the study had a very low response rate (5%). The recruitment strategies adopted in this study (large, non-personally tailored mail-out) have been used in previous studies of disadvantaged adults; however, generally they have been used in conjunction with other recruitment methods [46]. One recent study suggested that alternative recruitment strategies such as working with community leaders who can help seek out potential participants and using ‘word of mouth’ may be effective for recruiting socio-economically disadvantaged populations [47]. However, further studies are needed to identify the most effective recruitment strategies for reaching such a high-risk and hard-to-reach target group.

### Conclusions

The findings of the current study have potential implications for physical activity and sedentary behavior intervention research amongst women living in socioeconomically disadvantaged neighborhoods. An individually-tailored, locally relevant and multi-component approach may be feasible for increasing physical activity and reducing sedentary behavior amongst this target group. However, intervention studies are needed to test the effectiveness of the proposed strategies. Further, increasing promotion and education about the independent health risks linked to engaging in long periods of sedentary behavior, as well as strategies to reduce sedentary behavior, may be important considerations for future health promotion research amongst women living in disadvantaged neighborhoods.
